# Epigenotype–genotype–phenotype correlations in *SETD1A* and *SETD2* chromatin disorders

**DOI:** 10.1093/hmg/ddad079

**Published:** 2023-05-11

**Authors:** Sunwoo Lee, Lara Menzies, Eleanor Hay, Eguzkine Ochoa, France Docquier, Fay Rodger, Charu Deshpande, Nicola C Foulds, Sébastien Jacquemont, Khadije Jizi, Henriette Kiep, Alison Kraus, Katharina Löhner, Patrick J Morrison, Bernt Popp, Ruth Richardson, Arie van Haeringen, Ezequiel Martin, Ana Toribio, Fudong Li, Wendy D Jones, Francis H Sansbury, Eamonn R Maher

**Affiliations:** Department of Medical Genetics, University of Cambridge, Cambridge CB2 0QQ, UK; Department of Clinical Genetics, Great Ormond Street Hospital, London WC1N 3JH, UK; Department of Clinical Genetics, Great Ormond Street Hospital, London WC1N 3JH, UK; Department of Medical Genetics, University of Cambridge, Cambridge CB2 0QQ, UK; Department of Medical Genetics, University of Cambridge, Cambridge CB2 0QQ, UK; Stratified Medicine Core Laboratory NGS Hub, Department of Medical Genetics, University of Cambridge, Cambridge Biomedical Campus, Cambridge, UK; Department of Medical Genetics, University of Cambridge, Cambridge CB2 0QQ, UK; Stratified Medicine Core Laboratory NGS Hub, Department of Medical Genetics, University of Cambridge, Cambridge Biomedical Campus, Cambridge, UK; Manchester Centre for Genomic Medicine, Manchester University Hospitals NHS Foundation Trust, Saint Mary’s Hospital, Manchester, UK; Wessex Clinical Genetics Services, University Hospital Southampton NHS Foundation Trust, Southampton, UK; CHU Sainte-Justine Research Centre, Montreal, Quebec, Canada; Department of Pediatrics, University of Montreal, Montreal, Quebec, Canada; CHU Sainte-Justine Research Centre, Montreal, Quebec, Canada; Department of Neuropediatrics, University Hospital for Children and Adolescents, Leipzig, Germany; Yorkshire Regional Genetics Service, Chapel Allerton Hospital, Leeds, UK; Department of Genetics, University of Groningen, University Medical Center Groningen, Groningen, The Netherlands; Patrick G Johnston Centre for Cancer Research and Cell Biology, Queens University Belfast, Belfast, UK; Institute of Human Genetics, University of Leipzig Medical Center, Leipzig, Germany; Center of Functional Genomics, Berlin Institute of Health at Charité, Universitätsmedizin Berlin, Berlin, Germany; Northern Genetics Service, The Newcastle upon Tyne Hospitals NHS Foundation Trust, Newcastle, UK; Department of Clinical Genetics, Leiden University Hospital, Leiden, The Netherlands; Department of Medical Genetics, University of Cambridge, Cambridge CB2 0QQ, UK; Stratified Medicine Core Laboratory NGS Hub, Department of Medical Genetics, University of Cambridge, Cambridge Biomedical Campus, Cambridge, UK; Department of Medical Genetics, University of Cambridge, Cambridge CB2 0QQ, UK; Stratified Medicine Core Laboratory NGS Hub, Department of Medical Genetics, University of Cambridge, Cambridge Biomedical Campus, Cambridge, UK; MOE Key Laboratory for Cellular Dynamics, The School of Life Sciences, Division of Life Sciences and Medicine, University of Science and Technology of China, Hefei, Anhui 230026, China; Department of Clinical Genetics, Great Ormond Street Hospital, London WC1N 3JH, UK; All Wales Medical Genomics Service, NHS Wales Cardiff and Vale University Health Board and Institute of Medical Genetics, University Hospital of Wales, Heath Park, Cardiff, UK; Department of Medical Genetics, University of Cambridge, Cambridge CB2 0QQ, UK

## Abstract

Germline pathogenic variants in two genes encoding the lysine-specific histone methyltransferase genes *SETD1A* and *SETD2* are associated with neurodevelopmental disorders (NDDs) characterized by developmental delay and congenital anomalies. The *SETD1A* and *SETD2* gene products play a critical role in chromatin-mediated regulation of gene expression. Specific methylation episignatures have been detected for a range of chromatin gene-related NDDs and have impacted clinical practice by improving the interpretation of variant pathogenicity. To investigate if *SETD1A* and/or *SETD2*-related NDDs are associated with a detectable episignature, we undertook targeted genome-wide methylation profiling of > 2 M CpGs using a next-generation sequencing-based assay. A comparison of methylation profiles in patients with *SETD1A* variants (*n* = 6) did not reveal evidence of a strong methylation episignature. A review of the clinical and genetic features of the *SETD2* patient group revealed that, as reported previously, there were phenotypic differences between patients with truncating mutations (*n* = 4, Luscan-Lumish syndrome; MIM:616831) and those with missense codon 1740 variants [p.Arg1740Trp (*n* = 4) and p.Arg1740Gln (*n* = 2)]. Both *SETD2* subgroups demonstrated a methylation episignature, which was characterized by hypomethylation and hypermethylation events, respectively. Within the codon 1740 subgroup, both the methylation changes and clinical phenotype were more severe in those with p.Arg1740Trp variants. We also noted that two of 10 cases with a *SETD2*-NDD had developed a neoplasm. These findings reveal novel epigenotype–genotype–phenotype correlations in *SETD2-*NDDs and predict a gain-of-function mechanism for *SETD2* codon 1740 pathogenic variants.

## Introduction

Precise epigenetic regulation of gene expression is critical for normal human development (1). In the past two decades, increasing numbers of developmental disorders have been found to result from pathogenic variants in genes with important roles in chromatin structure and/or function or in DNA methylation (2–5). These epigenetic developmental disorders were initially delineated by clinicians who recognized a specific clinical phenotype often consisting of combinations of neurodevelopmental delay, congenital defects and characteristic facial dysmorphisms. With the advent of high-throughput genome-wide sequencing technologies, there has been a marked expansion in the number of neurodevelopmental disorders that are known to result from variants in epigenetic regulators (6–8). In addition, it has become clear that (a) specific clinical syndromes may result from pathogenic variants in multiple genes and (b) the phenotypic spectrum of some epigenetic disorders is wider than originally proposed, for instance, some isolated neurodevelopmental disorders may result from variants in genes previously linked to a specific syndrome (7,9–12). These developments can complicate the interpretation of variants of uncertain significance (VUS) in NDD genes, such that the absence of a classical phenotype may not be enough to exclude pathogenicity. Recently, it has been recognized that many epigenetic developmental disorders are associated with altered methylation profiles (episignatures) in peripheral blood (3). This observation has opened new approaches to aid VUS interpretation and investigate genotype–phenotype correlations and, to date, episignature alterations have been described in >50 distinct epigenetic disorders (13).

Enzymatic modification of amino acids within histone tails is a critical process for epigenetic regulation. Histone lysine methyltransferases may be divided into two classes: those containing the SET domain and those that lack the SET domain (14). Genes encoding SET domain containing lysine-specific histone methyltransferases include genes encoding histone H3 lysine 4 (KMT2) methyltransferases (KMTs), Nuclear receptor binding SET Domain protein 1 (*NSD1*) and *SET* genes (e.g. *SETD1A, SETD1B* and *SETD2*). Pathogenic variants in *KMT2* genes are associated with epigenetic neurodevelopmental disorders including Wiedemann-Steiner syndrome (MIM:605130; *KMT2A*), childhood dystonia 28 (DYT-KMT2B, MIM:617284; *KMT2B*), Kleefstra syndrome type 2 (MIM:617768; *KMT2C*), Kabuki syndrome type 1 (MIM:147920; *KMT2D*) and O’Donnell-Luria-Rodan syndrome (MIM:618512, *KMT2E*) that have overlapping but distinct phenotypes and distinct episignatures (2,5,15–22). Heterozygous pathogenic variants in *NSD1* are associated with Sotos syndrome (MIM:117550) which is characterized by pre- and post-natal overgrowth, macrocephaly, facial dysmorphisms, developmental delay and tumor susceptibility (20,21). Pathogenic variants in *SETD1A* have been reported in association with early onset epilepsy, neurodevelopmental delay and an increased risk of schizophrenia (MIM:618832; MIM:619056), and variants in *SETD1B* (MIM:619000), *SETD2* and *SETD5* (MIM:616761) have also been associated with NDDs (22–26). The target lysines may differ between different SET-domain-containing proteins, for instance, H3K4 (histone 3 lysine 4) for *SETD1A* and H3K36 (histone 3 lysine 36) for *SETD2* and *NSD1* (27)*.* Interestingly, *SETD2* variants have been associated with a range of clinical phenotypes. Germline heterozygous *SETD2* pathogenic variants were first described in association with a Sotos-like congenital overgrowth disorder associated with macrocephaly, intellectual disability, autism and obesity [known as Luscan-Lumish syndrome (MIM:616831)] (7,28–31). More recently a recurrent missense substitution [c.5218C > T (p.Arg1740Trp/R1740W)] was described in association with phenotypes of global developmental delay, failure to thrive and feeding difficulties, microcephaly (now known as Rabin-Pappas syndrome; MIM:620155) that was distinct from Luscan-Lumish syndrome plus a milder phenotype relating to a c.5219G > A (p.Arg1740Gln/R1740Q) variant (25).

To gain further insights into genotype–phenotype correlations and potential epigenotype alterations in *SETD1A* and *SETD2*-related neurodevelopmental disorders, we undertook genome-wide methylation profiling studies of ~2 M CpGs in these conditions and assessed the methylation episignatures of these disorders.

## Results

### Genotype–phenotype correlations

The details of the germline variants in *SETD1A* (*n* = 6) and *SETD2* (*n* = 10) are listed in Tables 1 and 2. Three variants were recurrent: *SETD1A* [c.4582-2_4582-1del (*n* = 3 individuals), *SETD2* (c.5219G > A (R1740Q, *n* = 2) and *SETD2* c.5218C > T (R1740W, *n* = 4)]. The frequency of the clinical features in individuals with *SETD2* variants is summarized in Table 2. The patient group with *SETD2* variants was subdivided into those three subgroups; based on previously published genotype–phenotype correlations from Rabin *et al.* (25): four individuals with truncating variants (*SETD2*-LLS-PX subgroup) and two groups with missense substitutions at codon 1740; Type 1 (R1740W, *n* = 4) and Type 2 (R1740Q, *n* = 2). As described previously, all individuals with *SETD2* LoF variants displayed macrocephaly and mild to moderate intellectual disability. In contrast, those with codon 1740 missense substitutions demonstrated microcephaly rather than macrocephaly and microcephaly, severe intellectual disability, congenital anomalies (renal, cardiac and central nervous system) and a vascular retinopathy were present in Type 1 (R1740W) subgroup (see Table 2). A further feature of the *SETD2*-cohort was the occurrence of tumors in two of 10 individuals. A patient with a *SETD2* LOF variant developed multiple brain stem gliomas (*SETD2*-LLS-P2) from age 6 years and a patient (*SETD2*-R1740W-P4) with Type 1 (R1740W) variant with a metastatic high-grade chondroblastic osteosarcoma in her right proximal tibia and right mandible age 15 years. It was not possible to say which was the primary lesion and following a decision for palliative treatment, she died shortly after presentation. In addition, a further patient (*SETD2-*R1740W-P2) was previously reported to have had a hypothalamic hamartoma (25). The frequency of the clinical features in individuals with SETD1A variants is summarized in Table 2.

**Table 1:** *SETD2* and *SETD1A* variant details

Sample ID	Variant	Age/sex	Previously published	ACMG-AMP criteria
*SETD2-R1740Q-P1*	c.5219G > A p.(Arg1740Gln) *de novo* missense	12/F		PM1, PM2, PM6, PP3
*SETD2-R1740Q-P2*	c.5219G > A p.(Arg1740Gln) *de novo* missense	5/M		“
*SETD2-R1740W-P1*	c.5218C > T p.(Arg1740Trp) *de novo* missense	0.5/M	Patient 7 (25)	PM1, PM2, PM6, PP3
*SETD2-R1740W-P2*	c.5218C > T p.(Arg1740Trp) *de novo* missense	10/M	Patient 11 (25)	“
*SETD2-R1740W-P3*	c.5218C > T p.(Arg1740Trp) *de novo* missense	10/F	Patient 9 (25)	“
*SETD2-R1740W-P4*	c.5218C > T p.(Arg1740Trp) *de novo* missense	12/F	Patient 4 (25)	“
*SETD2-LLS-P1*	c.4438_4441del p.(Val1480fs) *de novo* frameshift	12/M		PVS1, PM2, PM6, PP3
*SETD2-LLS-P2*	c.1647_1667delinsTG p.(Ser550AspfsTer23) *de novo* frameshift	4.5/M	Case 1 (30)	PVS1, PM2, PM6, PP3
*SETD2-LLS-P3*	c.513del p.(Pro172fs) *de novo* frameshift	2/M		PVS1, PM2, PM6, PP3
*SETD2-LLS-P4*	c.4457_4460delAGAA p.(Lys1486ArgfsTer28) *de novo* frameshift	20/F		PVS1, PM2, PM6, PP3
*SETD1A-P1*	c.4582-2_4582-1del *de novo* splice acceptor	3/M		“
*SETD1A-P2*	c.4582-2_4582-1del *de novo* splice acceptor	17/F		“
*SETD1A-P3*	c.4582-2_4582-1del *de novo* splice acceptor	13/F		“
*SETD1A-P4*	c.4711C > T p.(Arg1571Ter) Nonsense	3/F		PVS1, PM2, PP3
*SETD1A-P5*	c.2289dup p.Val764Serfs^*^61 *de novo* frameshift	7/F		PVS1, PM2, PM6, PP3
*SETD1A-P6*	Whole gene deletion CNV (852 kb loss of 41 genes)	12/F		PVS1, PM2, PP3

**Table 2:** Frequency (%) of clinical features in the three subgroups of patients with pathogenic variants in *SETD2*

	*SETD2* variant subgroups		
	R1740Q^a^	R1740W^a^	Loss of Function variants^a^
Number of patients	2	4	4
Clinical Features			
Tall stature (HP:0000098) (%)	0	0	25%
Obesity HP:0001513 (%)	50	0	50
Macrocephaly (HP:0000256) (%)	0	0	100
Microcephaly (HP:0000252) (%)	0	100	0
Retinopathy (HP:0000488) with retinal hemorrhage and/or detachment (%)	0	100	0
Micrognathia HP:0000347 (%)	0	50	0
Failure to thrive (HP:0001508) requiring nasogastric feeding (%)	0	50	0
Renal anomaly (HP:0000077) (cystic dysplasia or dilation renal pelvis) (%)	0	100	0
Congenital heart anomaly HP:0001627) (%)	0	100	25
Scoliosis HP:0002650 (%)	0	50	25
Severe developmental delay (HP:0012758) (%)	0	100	0
Mild/moderate developmental delay (HP:0012758) (%)	100	0	100
Seizures (HP:0001250) (%)	0	50	0
CNS structural anomaly (HP:0002011) (%)	0	100	25
Neoplasm (HP:0002664) (%)	0	25	25

Detailed descriptions of the SETD2-R1740W patient phenotypes are included in Rabin *et al*. (25).

^a^R1740Q = c.5219G > A p.(Arg1740Gln). R1740W = c.5218C > T p.(Arg1740Trp). Loss of Function variants = frameshift variants.

**Table 3:** Frequency (%) of clinical features in the three subgroups of patients with pathogenic variants in *SETD1A*

	% with clinical feature
Number of patients	6
Clinical features	
Macrocephaly (HP:0000256) (absolute or relative) (%)	83
Short stature (HP:0004322) <10th centile (%)	33
Non-specific facial dysmorphisms HP:0001999 (%)	100
Joint laxity (HP:0001388) or hypermobility (%)	50
Sleep disturbance (HP:0002360) (%)	66
Brain MRI anomalies (HP:0002011) (%)	17
Mild—moderate neurodevelopmental delay (HP:0012758) (%)	83
Severe—profound neurodevelopmental delay (HP:0012758) (%)	17
Autistic spectrum features (HP:0000729) (%)	50
Seizures (HP:0001250) (%)	17
Congenital anomalies	0

### Epigenotype–genotype–phenotype analysis

**Figure 1:**
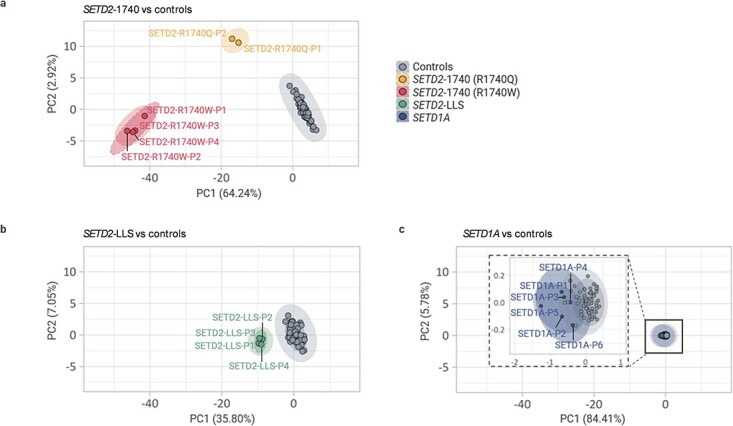
Clustering of *SETD2* and *SETD1A* based on methylation episignatures. Unsupervised PCA clustering results for *SETD1A* and *SETD2* group. Sample name and group annotations were applied by each group after PCA. Dotted line: group names annotation, Solid line: clustering by ‘stat_ellipse’ function in R (assumes a multivariate t-distribution; applied except for the Type 2 (R1740Q) group since this function applies only when there are more than two samples in a group). (**A**) There were two distinct groups in *SETD2* NDD patient samples. There was a significant difference between *SETD2*-1740 samples and controls but Type 2 (R1740Q) cases were closer to controls than Type 1 (R1740W). (**B**) Despite being distinct from controls, the distance of *SETD2*-LLS cases were much closer to the control group than *SETD2*-1740 Type2 (R1740Q) cases. (**C**) *SETD1A* cases were not distinguishable from the healthy controls. There are no detectable differences between the two groups of *SETD1A* splice acceptor variants (*SETD1A*-P1, P2, P3) or pathogenic variants (*SETD1A*-P4, P5, P6).

*Principal component analysis (PCA) of DNA methylation profiles*: The PCA (unsupervised clustering) of significant CpG sites after filtering was performed separately for individuals with *SETD1A* NDD and *SETD2* NDD (Fig. 1). *SETD1A* samples with pathogenic variants (*n* = 6) were not distinguishable from the control cohort (Fig. 1C) and subclassifying the *SETD1A* NDD samples into those with the recurrent *SETD1A* splice acceptor variant (*n* = 3) and other cases with pathogenic variants (*n* = 3) (frameshift, stop gained and CNV) did not show any detectable difference between the two groups (Fig. 1C). Comparison of *SETD2* NDD patient samples and controls separated those with loss of function variants and those with codon 1740 missense variants (Fig. 1A and B). Based on previous genotype–phenotype correlations (25), the *SETD2*-1740 group were subdivided into Type 1 (R1740W) and Type 2 (R1740Q) and when compared with non-1740 pathogenic variants (*SETD2*-LLS) each subgroup was distinct from controls (Fig. 1B). The methylation episignatures were then interrogated for individual *SETD1A* and *SETD2* NDD individuals.

**Figure 2:**
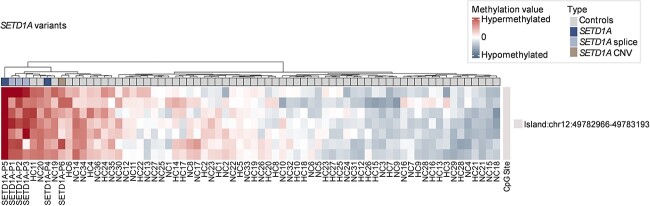
Methylation episignatures for *SETD1A* samples. *SETD1A* NDD did not exhibit a prominent methylation episignature. The hierarchical clustering was not able to separate *SETD1A* from control groups. A comparison of methylation profiles in 6 patients with a *SETD1A* NDD to those in 64 controls. Methylation analysis detected 7 significant differentially methylated CpG positions with 1 CpG island (chr12:49782966-49783 193) and no DMBs were detected.

*SETD1A NDD episignatures*: Comparison of methylation profiles in 6 individuals with a *SETD1A* NDD to those in 64 controls identified 7 significant differentially methylated CpG positions (DMPs) with 1 CpG island (chr12:49782966-49783193) and no DMBs detected (shown in Fig. 2). Using similar methodology, we previously found that comparison of methylation profiles in *KMT2B* NDD individuals (*n* = 10) and *KMT2D* associated Kabuki syndrome individuals (*n* = 10) to control samples (*n* = 29) identified 1812 and 89 significant DMPs, respectively (5). From the results of in-house laboratory healthy controls quality control, random sampling of the methylation profiles from 64 normal control samples for a range of 1 to 15 normal controls showed a mean 3.6 (range 0 to 17) significant CpGs. Therefore, there was no evidence of a strong methylation episignature in the *SETD1A* NDD group and, as shown in Figure 2, the hierarchical clustering was unable to clearly distinguish *SETD1A* from control groups.

The methylation profiling of *SETD1A* samples (*n* = 3) with the recurrent splice-acceptor variant were then compared with normal controls but this identified only 17 significant DMPs with 2 DMBs and 1 CpG island (shown in Supplementary Material, Fig. S1). The small number of significant DMBs found in the *SETD1A* cohort was consistent with no strong methylation episignature. As shown in Figure 2B, the hierarchical clustering was unable to clearly distinguish *SETD1A* from control groups. A CpG island (GRCh37:Chr12:49782966-49783193) that passed the filter is associated with the *SPATS2* (spermatogenesis associated serine-rich 2) gene, but no other genes or ClinGen diseases have been identified in this region as of yet.

**Figure 3:**
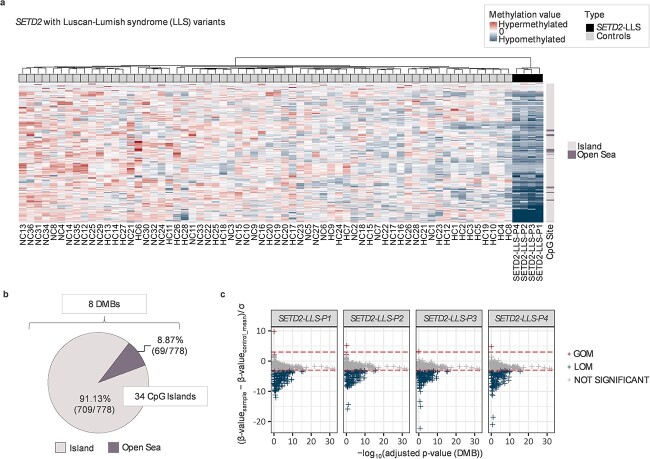
Methylation episignatures for *SETD2*-LLS samples. The methylation episignatures for *SETD2*-LLS LoF variant patients (*n* = 4) displayed 778 DMPs (767 hypomethylated and 11 hypermethylated), including 34 CpG Islands and 8 DMBs. (**A**) Hierarchical clustering on the top annotation bar revealed that 4 *SETD2*-LLS patients have remarkable hypomethylated profiles that are distinguishable from control samples. (**B**) The majority of DMPs detected as significant are CpG islands (91.13%), whereas the rest of the DMPs (69 DMPs within 8 DMBs) are located in the Open Sea area (i.e. the rest of the genomic regions except Shelf, Shore or CpG Islands). (**C**) Normalized methylation values were used to determine whether methylated DMPs gained or lost methylation. The horizontal line (red) indicates a confidence interval of 3 standard deviations (3SD). As a result, all four patients (all of whom are frameshift variants) had similar LOM patterns of methylation. ^*^DMB: Differentially methylated blocks ^*^GOM: Gain of methylation ^*^LOM: Loss of methylation.

**Figure 4:**
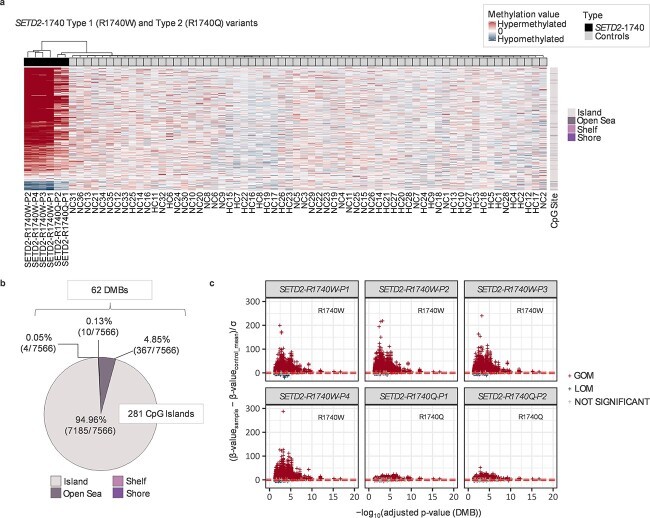
Methylation episignatures for *SETD2*-1740 samples. The *SETD2*-1740 samples (*n* = 6) are revealed to have extensive methylation alterations with 7566 significant DMPs (789 hypomethylated and 6777 hypermethylated) containing 281 CpG Islands and 62 DMBs. (**A**) Hierarchical clustering dendrogram revealed that the majority of significant DMPs in *SETD2*-1740 cases exhibited very clear hypermethylation patterns compared with controls. A noteworthy observation is that 4 *SETD2*-1740 patients with Type 1 (R1740W) are distinguishable from Type 2 cases (R1740Q) and healthy controls. (**B**) Of the 7566 DMPs, 94.96% of DMPs are located on CpG islands, followed by Open Sea (4.85%), Shore (0.13%), and Shelf (0.05%). (**C**) Gain or loss of methylated DMPs were determined by the normalized methylation values. The horizontal line (red) indicates a confidence interval of 3 standard deviations (3SD). As a result, all four Type 1 (R1740W) cases showed a more severe hypermethylation pattern than in the two Type 2 (R1740Q) cases. ^*^DMB: Differentially methylated blocks ^*^GOM: Gain of methylation ^*^LOM: Loss of methylation.

*SETD2-1740 and SETD2-LLS NDD cohorts:* In contrast to the *SETD1A* NDD individuals, a combined analysis of the methylation profiles in 10 individuals with *SETD2* variants to controls (*n* = 64) identified 135 significant DMPs (17 hypomethylated and 118 hypermethylated for *SETD2*-1740, 81 hypomethylated and 54 hypermethylated for *SETD2*-LLS) with 1 DMB and 5 CpG islands (Supplementary Material, Fig. S2). Furthermore, when the methylation profiles of *SETD2*-1740 samples and non-*SETD2*-1740 samples were analyzed separately, there were clearer and distinct, methylation episignatures in both groups (Figs 3 and 4). Methylation episignatures for non-*SETD2-*1740 (LoF) variant individuals (*n* = 4) displayed 778 DMPs (767 hypomethylated and 11 hypermethylated), including 34 CpG Islands and 8 DMBs (Fig. 3).

The *SETD2*-1740 samples (*n* = 6) demonstrated more extensive methylation alterations with 7566 significant DMPs (789 hypomethylated and 6777 hypermethylated) containing 281 CpG Islands and 62 DMBs (Fig. 4). Thus, there were both quantitative and qualitative differences in the methylation episignatures between *SETD2* LoF variant samples and *SETD2*-1740 missense substitution individuals with the majority of significant DMPs in *SETD2-*1740 cases exhibiting hypermethylation compared with controls. Previously, it has been observed that there are phenotypic differences between Type 1 (R1740W) and Type 2 (R1740Q) cases (R1740W and R1740Q, respectively) that Type 1 (R1740W) having a more severe phenotype. Episignature results showed that all four Type 1 cases showed a more severe hypermethylation pattern than in the two Type 2 (R1740Q) cases (Fig. 4A and 4C). In addition, as alterations at individual DMPs may correlate with alterations at other DMPs, we used the caret package (Classification And Regression Training) to identify non-redundant DMPs and identified 139 DMPs for *SETD2*-1740 and 60 DMPs for *SETD2*-LLS group (see Supplementary Material, Tables S1 and S2) (32).

Comparative analysis for *SETD2*-1740 and *SETD2*-LLS cases confirmed that methylation episignatures of *SETD2*-LLS were less distinctive than *SETD2*-1740 cases (shown in Supplementary Material, Fig. S2). Of the 7566 significant DMPs in the *SETD2-*1740 cases, 513 DMPs (include 25 Islands and 4 DMBs) were also significant in non-*SETD2-*1740 (LoF variant) samples (see Supplementary Material, Fig. S3). In the *SETD2*-LLS group, over half of the significant CpG islands and DMBs overlapped with the *SETD2*-1740 group. In order to investigate if the overlapped CpGs might contribute to their certain overlapped phenotypes (whereas those that are not overlapped may provide insight into their distinct phenotypes), we examined the genes and pathways affected by methylation changes in target genes.

### Gene and pathway analysis for target gene methylation alterations

**Figure 5:**
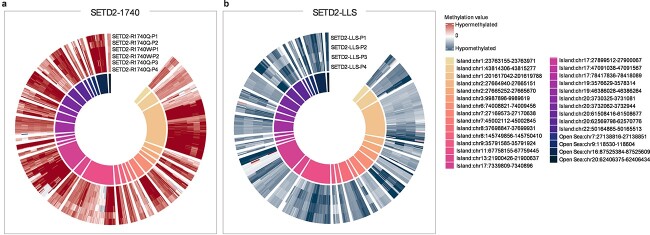
Methylation episignatures for overlapped CpGs in *SETD2* patients. Common CpGs between *SETD2*-1740 and *SETD2*-LLS. Overlapped DMPs were identified from the data in Figure 2 (*SETD2*-1740 versus control) and Figure 3 (*SETD2*-LLS versus control) (NOT based on a comparative analysis from Supplementary Material, Fig. S3). Several overlapped DMPs are summarized in Supplementary Material, Figure S4A and SB. There are 25 CpG islands and 4 DMBs overlapped between the two groups. All DMPs show (**A**) hypermethylated across 6 *SETD2*-1740 cases [Type 2 (R1740Q) cases display slightly milder episignatures than Type 1 (R1740W)] whereas (**B**) all hypomethylated in 4 *SETD2*-LLS cases. The episignatures of both groups were completely opposite.

The 7566 DMPs in *SETD2*-1740 were associated with 362 genes whereas the DMPs in non-*SETD2*-1740 individuals were associated with 43 genes (29 genes were common to both *SETD2*-1740 and non-*SETD2*-1740 cases) (Supplementary Material, Table S3). Given the phenotypic differences between the two subgroups of *SETD2* NDD, we compared the biological pathways for genes associated with *SETD2*-1740 only, non-*SETD2*-1740 only and both patient groups (see Supplementary Material, Tables S4–S6). A clear phenotypic difference between *SETD2*-1740 and non-*SETD2*-1740 individuals is the occurrence of growth retardation in the former and frequent overgrowth in the latter. Genes implicated in growth control associated with DMPs included *NRP2, LTBP3, MRPS34, RAI1, SUCLA2, IGF2BP1* and *IGF1R* in the *SETD2*-1740 cases. Among them, *NRP2* and *IGF2BP1* were identified in non-*SETD2*-1740 individuals as well. Neither of these genes has been confirmed to cause overgrowth or growth retardation in the past. However, methylation episignature results showed that both *NRP2* (chr9:35791585-35791924) and *IGF2BP1* (chr17:47091038-47091567) were associated with hypermethylated CpG islands in *SETD2*-1740 but hypomethylated CpG islands in non-*SETD2*-1740. Additionally, all overlapped DMPs between two *SETD2* groups showed the opposite methylation profiles (Fig. 5).

Somatic *SETD2* mutations occur in a range of human cancers, in particular clear cell renal cell carcinomas (ccRCCs). None of the DMP-associated genes in *SETD2* NDD individuals were frequent targets for somatic mutations in ccRCC, but 51 DMP-associated genes have been linked to oncogenesis (details supplied in ‘NCG7.0’ tab from Supplementary Material, Tables S4–S6). Among non-*SETD2*-1740 individuals, 7 genes (*MPL, NAV1, PRRT3, MYO1G, ELMO3, NLRP3* and *KLHDC4*) were classified as cancer genes from the Network of cancer genes database (http://ncg.kcl.ac.uk/query.php). Among them, *MPL* (chr1:43814306-43815277), *NAV1* (chr1:201617042-201619788), *PRRT3* (chr3:9987896-9989619) and *MYO1G* (chr7:45002112-45002845) were also identified in altered DMPs in *SETD2*-1740 individuals. *MPL* (proto-oncogene), *NAV1* and *PRRT3* are putative oncogenes implicated as candidate cancer drivers. *MYO1G* is a putative tumor suppressor. All overlapped genes were associated with hypermethylation in *SETD2*-1740 and hypomethylation in non-*SETD2*-1740 (see Fig. 5).

## Discussion

We investigated potential methylation episignatures in individuals with germline variants in the lysine methyltransferase *SETD1A* and *SETD2* genes using a targeted bisulfite sequencing-based approach that profiles ~2 M CpG sites across the genome. Though we did not detect evidence for a strong episignature for *SETD1A*, we found distinctive epigenotype–genotype–phenotype correlations for *SETD2-*associated NDDs. To our knowledge, investigations of the episignature of *SETD1A* and *SETD2* NDDs have not been reported previously with an NGS-based methylation profiling strategy but a methylation array-based episignature has been reported for *SETD2* (LLS) and *SETD1B*- related NDD (13,33). We note that in the *SETD2*-LLS methylation array-based episignature from Levy *et al*. (4,13) loss of methylation alterations predominanted (similar to that in our NGS-based *SETD2*-LLS methylation episignature with hypomethylated patterns) and further comparison between the episignatures is provided in Supplementary Material, Table S7. Although a variety of investigative options are available for genome-wide methylation profiling, the most commonly applied are Illumina methylation arrays that can analyze 450 000 or ~850 000 CpGs. Previously we described the use of the Illumina TruSeq Methyl Capture Library kit and next-generation sequencing (EPIC-NGS) to detect methylation episignatures for *KMT2B*-associated dystonia and Type 1 and *KMT2D*-related Kabuki syndrome, confirming the results of methylation profiles for these disorders using methylation arrays (5,34). To validate the specificity of *SETD2*-1740 and *SETD2*-LLS methylation episignatures compared with those of *KMT2B*-related childhood-onset dystonia and *KMT2D*-related Kabuki syndrome type 1 cohort, unsupervised clustering was performed and 29 significant DMBs could successfully discriminate *SETD2* NDDs from other NDDs (see Supplementary Material, Figs S4 and S5 and Supplementary Material, Table S8).

More than 50 human disorders have been investigated for methylation episignatures [reviewed by Levy *et al.* (13)] and episignatures have been described for many of these. However, the strength and specific patterns of methylation alterations vary between disorders. Though we did not identify a robust episignature for *SETD1A* NDD using an approach that interrogates more than twice the number of CpGs as methylation array-based methods, there is little information regarding the relative performance of the two techniques and we cannot exclude that an episignature may have been detectable with another methodology and/or a larger number of individuals with pathogenic variants. However, we suggest that if there is a methylation episignature for *SETD1A* NDD then it is less pronounced for *SETD2*, *KMT2B* and *KMT2D*-associated disorders. In addition, it should be noted that our results relate to the methylome of peripheral blood and while this is a standard approach for the investigation of epigenetic disorders caused by pathogenic variants in chromatin-modifying genes, we cannot exclude the possibility that a *SETD1A* methylation episignature might vary between tissues and be more prominent in target organs such as the central nervous system (mosaic epimutations with variable levels in different tissues are well-recognized in epigenetic disorders associated with disordered genomic imprinting (35). Finally, we note that while *SETD1A* and *SETD2* target different histone lysines (H3K4 and H3K36 respectively), *KMT2B* and *KMT2D* also act on histone 3 lysine 4 (H3K4).

In contrast to *SETD1A* NDD, the results for *SETD2* NDD revealed clear methylation episignatures correlated with the phenotypic heterogeneity described previously in *SETD2-*associated NDDs. Thus, *SETD2*-1740 patterns of methylation differed both from controls and those with non-*SETD2*-1740 LoF variants in both the extent of methylation alterations (7566 and 778 significant DMPs respectively) but also the direction of methylation alterations with predominant hypermethylation in *SETD2*-1740 and predominant hypomethylation in non-*SETD2*-1740 variants. Furthermore, though the number of individuals were small, within the *SETD2*-1740 subgroup, there was an apparent difference between Type 1 (R1740W) and Type 2 (R1740Q) individuals with predominant hypermethylation alterations in both groups but with more pronounced alterations in Type 1 cases. In an analysis of 15 individuals with missense substitutions at codon 1740 (12 with R1740W and 3 with R1740Q), recently Rabin *et al.* (25) contrasted the phenotype of Type 1 (R1740W) individuals from that of 12 individuals with non-1740 variants (mostly truncating) who presented with autistic spectrum disorder (ASD) or an LLS, a Sotos-like NDD that is characterized by macrocephaly, overgrowth or obesity, ASD and variable intellectual disability but without congenital malformations of internal organs (25). In contrast, individuals in the Type 1 (R1740W) group were characterized by severe intellectual disability, CNS malformations, microcephaly, failure to thrive and multiple congenital malformations (e.g. congenital heart defects and urogenital anomalies) (25). Interestingly, individuals in Type 2 (R1740Q) group exhibited a similar, but milder, phenotype to Type 1 (R1740W) cases with intellectual disability and a small head circumference but without significant congenital anomalies. Therefore, although larger numbers of cases are required for confirmation, the more extensive methylation changes in Type 1 (R1740W) than in Type 2 (R1740Q) individuals do correlate with phenotypic severity.

In contrast to individuals diagnosed with LLS, cases with *SETD2*-1740 missense substitutions do not exhibit significant overgrowth. Interestingly, the methylation episignatures of LLS individuals and of other non-imprinting epigenetic disorders associated with congenital overgrowth such as Sotos syndrome and *DNMT3A*-overgrowth syndrome (Tatton-Brown-Rahman syndrome; MIM:615879) demonstrate predominant hypomethylation (36,37). A number of growth-related genes were associated with significantly altered DMPs in the current study e.g. the insulin-like growth factor receptor (*IGF1R*) gene, which was hypermethylated in the *SETD2*-1740 group. Germline mutations in *IGF1R* are associated with pre- and post-natal growth retardation and microcephaly (38) and combined investigations of the epigenetic and transcriptional effects of germline *SETD2* LoF and codon 1740 mutations could provide candidate genes for aspects of *SETD2*-associated NDDs. Moreover, we have found that among detected genes related to hypo- or hypermethylation patterns in *SETD2*-1740 and *SETD2*-LLS group (Supplementary Material, Tables S4 and S5), several genes related to NDDs are up-regulated (3 genes including *MRPS34*) and down-regulated (12 genes including *PLA2G6* and *MBOAT7*) in *SETD2* knockout mice model (Setd2^mNul^ oocyte) compared with healthy controls from GSE112835 (39) (see ‘differentially expressed genes from GSE112835’ tab in Supplementary Material, Tables S4 and S5). Our findings support that genes affected by aberrant methylation patterns could impact *SETD2* functioning, thereby could affect the phenotypes, although this may not be the primary cause since it is the second hit by aberrant methylation, and functional effects of *SETD2* deficiency may differ between mice and humans.

We note that two of 10 individuals with a *SETD2* NDD reported here had developed a tumor (a sarcoma in one case and multiple gliomas in the other). In addition, a further patient had had a CNS hamartoma. Tumor predisposition has been associated with other chromatin gene disorders associated with congenital overgrowth such as Sotos syndrome (e.g. overall risk ~ 3% including teratoma, neuroblastoma, ganglioma, leukemia, lung cancer and glioma) and Weaver syndrome (e.g. overall risk ~ 2% including leukemia, neuroblastoma and lymphoma) that are caused by mutations in the histone methyltransferases *NSD1* and *EZH2,* respectively (21,40). Furthermore, (a) *SETD2* is frequently mutated in a range of human cancers, with the highest frequencies in mesothelioma, endometrial cancer and renal cell carcinoma but somatic *SETD2* mutations also occur in sarcoma and glioblastoma multiforme (TCGA PanCancer Atlas) and (b) *SETD2* has been implicated in the repair of DNA double-strand breaks (41). Therefore, though neoplasia has not been reported previously in the published literature as a feature of a *SETD2* NDD, we are aware of an additional and unpublished case of a *SETD2* NDD patient with osteosarcoma (J. Bernat, personal communication) and this aspect requires further investigation and clinicians looking after these individuals should be aware of a possible causal association. However, at this stage, we would not recommend a tumor surveillance program until the tumor risks and types have been confirmed and the cost-effectiveness of a surveillance protocol could be more accurately predicted. We note that neoplasia occurred in both *SETD2*-1740 and non-*SETD2*-1740 patient groups. *SETD2* is a tumor suppressor gene and the most somatic driver *SETD2* mutations are truncating but the c.5218C > T (R1740W) somatic variant is listed five times (out of 2938 unique samples with mutations) entries in the Catalogue of Somatic Mutations in Cancer (COSMIC) database (accessed 5.01.2023) (the exact germline *SETD2* LLS-associated loss of function mutations described here are not present in the COSMIC database but a somatic mutation producing a very similar truncated protein (p.Lys1486Argfs^*^29) to that in *SETD2*-LLS-P4 has been reported in a single renal cell carcinoma (42). Therefore, despite evidence that germline truncating and Type 1 (R1740W) mutations have different effects on developmental phenotype and epigenotype, both can be oncogenic.

**Figure 6:**
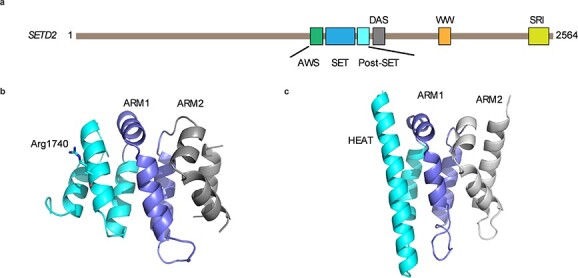
*SETD2* codon 1740 structural predictions. (**A**) Domain architecture of human *SETD2.* (**B**) AlphaFold model of *SETD2* DAS domain. (**C**) The structure of Iws1 conserved domain (PDB IDn2XPL).

Codon 1740 is not located in any known functional domain of the *SETD2* human protein, and maps to an all α-helix domain which is C-terminally adjacent to the post-SET domain (referred to as DAS domain, domain after SET) (Fig. 6A). Though the DAS domain does not map to a specific functional domain, it is highly conserved across multiple species (including *Danio rerio* and *Xenopus tropicalis*) suggesting functional importance. The AlphaFold model of *SETD2* (AF-Q9BYW2-F1) shows that the DAS domain can fold as a globular domain, further confirming its functional importance (Fig. 6B) (43). A further DALI search shows that the DAS domain structurally resembles the conserved region of chromatin remodeling factor Iws1 (44,45). The Iws1 conserved region is formed by a single HEAT repeat followed by two ARM repeats and has been shown to interact with an N-terminal region of Spt6 (Fig. 6C). This suggests that the DAS domain could act a protein–protein interaction module. Furthermore, from the AlphaFold model, Arg1740 is on the surface of the DAS domain, so the mutants, including R1740W and R1740Q, possibly disrupt the interaction network of the DAS domain, but not disrupt its overall folding (Fig. 6B). The observed differences in epigenotypes between *SETD2* truncating mutations *SETD2*-1740 missense substitutions would be consistent with a gain-of-function effect in the latter. Though the precise mechanisms of the effects of Type 1 (R1740W) and Type 2 (R1740Q) substitutions on *SETD2* function remain to be elucidated, the location within the DAS domain in a region predicted to function as a binding site for an unidentified protein provides a hypothesis that could be tested in further studies, as could whether the differences in observed phenotype severity for Type 1 (R1740W) and Type 2 (R1740Q) substitutions were correlated with differential effects on protein binding and gain-of-function effects.

In summary, we investigated genotype and epigenotype features of *SETD1A*- and *SETD2*-associated NDDs. We found evidence that *SETD2*-associated NDD may be associated with tumor susceptibility and, though we did not identify a clear methylation episignature for *SETD1A*-NDD, for *SETD2*-associated NDDs we identified methylation alterations that correlated with the clinical heterogeneity of *SETD2* codon 1740 and non-codon 1740 LoF mutations. These observations illustrate how methylation profiling and the detection of specific methylation episignature can provide insights into potential mechanisms of disease that might provide insights into possible approaches for the development of therapeutic interventions.

## Materials and Methods

### Study cohort

Genomic DNA from patients with germline *SETD1A* (*n* = 6) or *SETD2* (*n* = 10) variants were investigated. All variants were categorized as likely pathogenic or pathogenic according to ACMG-AMP criteria (https://wintervar.wglab.org/) (see Table 1). Details of patient ages at DNA sampling and sex are presented in Table 1. Locations of the *SETD2* (GRCh37/hg19, ENST00000409792.3; NM_014159.6) and *SETD1A* (GRCh37/hg19, ENST00000262519.8; NM_014712.1) variants on the respective proteins are shown in Supplementary Material, Fig. S6. For the individuals with a *SETD1A-*associated variant (1 male and 5 females) the mean age at DNA sampling was 9.2 years, whereas it was 8.8 years in the *SETD2-*NDD group (6 males and 4 females). Five *SETD2-*NDD patients had been described previously (*SETD2*-R1740W-P4*, SETD2*-R1740W-P1 *SETD2*-R1740W-P3 and *SETD2*-R1740W-P2 were patients 4, 7, 9 and 11, respectively, in Rabin *et al.* (25) and *SETD2*-LLS-P2 was Case 1 in van Rij *et al.* (30). Methylation profiling results from the *SETD1A-* and *SETD2-*NDD DNA samples were compared with those from 64 healthy control subjects (age range from 0 to 40 with 31 males and 33 females) (29 of whom were included in a previous publication) (5). All 64 controls were examined, and no significant age-related episignature was detected among them. Additionally, age, gender, and batch effects were corrected during differential methylation analysis. The study was performed in accordance with the Helsinki Declaration and written informed consent was obtained from parents/guardians of the patients and blood samples were collected under local study ethical approval. The study was approved by South Birmingham Research Ethics Committee.

### Molecular studies

DNA was extracted from whole blood by standard methods. DNA samples were quantified using the Qubit™ dsDNA BR Assay Kit (Invitrogen, ThermoFisher). DNA samples were sequenced on an Illumina NextSeq 2000 using a TruSeq^®^ Methyl Capture EPIC kit. Bisulfite conversion, library preparation, target enrichment and sequencing were performed at the Stratified Medicine Core Laboratory, Department of Medical Genetics of the University of Cambridge, as described previously (5).

### Data processing and bioinformatic analysis

The sequencing data were extracted in a FASTQ format. As a first step in filtering, sequencing reads with Phred score ≤ 30 were removed and adapter trimming steps were performed using Trim-Galore software (www.bioinformatics.babraham.ac.uk/projects/trim galore/). The trimmed sequences were subjected to FASTQC to ensure quality control. The sequenced reads were then aligned to the Genome Reference Consortium Human Build 37 (GRCh37) using the Bismark software v0.17.0 (www.bioinformatics.babraham.ac.uk/projects/bismark/) (46). Following alignment, PCR duplicates were removed using the ‘deduplicate bismark’ option. As a final step, methylation values (.bismarkcov format) were extracted using the ‘bismark methylation extractor’ function.

*Methylation analysis with RnBeads package:* Raw methylation beta-values and annotation information of CpG sites (Open Sea, Shelf, Shore and CpG Island), including the *P*-value of each CpG position were extracted using the Bioconductor RnBeads R package (https://rnbeads.org/). The RnBeads package was implemented in R software (available on R version 3.6.3). The Bismarkcov files were directly loaded into RnBeads as BED files. As part of the loading procedure, regions with X, Y chromosomes and CpGs that fall near a SNP were removed. The sequencing coverage threshold was set at 10X. CpG sites with exceptionally high coverage outliers and missing values present in more than one sample were removed during the filtering process. Differential methylation analysis option (by ‘*limma*’ method) was used for extracting beta-values and annotation (mean difference between groups, a *P*-value of each site) information (47,48). When computing differentially methylated CpG sites, PCA was performed to assess any batch effect (age, gender, batch) and identify significant outliers. If a significant batch effect was detected, the target variables were adjusted by surrogate variable analysis (SVA) using the sva package. Then, age, batch, and gender were considered as covariates when assessing differentially methylated regions by the *limma* method using the RnBeads ‘covariate.adjustment.columns’ option. Moreover, cell type heterogeneity is widely recognized as a source of confounding in DNA methylation profiling studies, thus an adjustment step using the Houseman method (49) was included in the pipeline to minimize the effects of variations in blood composition. Under the *limma* package, RnBeads offers cell type adjustment, which were enabled with the ‘differential.adjustment.celltype’ and ‘inference.reference.methylome.column’ = ‘Celltype’ options. By incorporating estimates of cell type proportions into the differential methylation analysis, differences in cell type proportions were adjusted, and any potential bias related to differences in cell type composition between samples could be eliminated, thus increasing the accuracy of the results.

As a result, three different output files were obtained: (1) mean difference between control and disease groups, (2) site *P*-value obtained from a two-sided Welch t-test or alternatively from linear models employed in the *limma* package and (3) combined *P*-value for CpG Islands using a generalization of Fisher’s method. The obtained data were used for the further differentially methylated block (DMB) and methylation episignature analysis. A summary of the sequencing coverage and sequencing reads is provided in Supplementary Material, Table S9. Data is available on request from the authors (subject to patient consent).

*Detection and visualization of methylation episignatures:* Only CpG sites (or CpG islands) with a neighboring CpG count greater than 5 kb were selected for further analysis. Neighboring CpGs located in CpG sites (Open Sea, Shore and Shelf) were then combined together and assigned as ‘DMB’. DMBs were combined on the basis of their functional similarity. Each DMB has a size ranging from 5 to 200 kb. On the other hand, CpG islands are determined by the Ensembl genome browser (http://www.ensembl.org) (48). Based on the discussed threshold, significant DMBs from *SETD2* and *SETD1A* cohorts were selected for methylation signature analysis. As a first filtering step, DMBs that contained fewer than three EPIC-NGS target regions were filtered out. After that, only DMBs (including CpG Islands) with an adjusted *P*-value lower than 0.05 (false discovery rate < 0.05) and a methylation difference between controls and diseases group of more than 20% were considered significant for genome-wide CpG site methylation analysis. Moreover, genes associated with significant DMBs and CpG islands, including OMIM/Morbid association, were annotated using the DECIPHER browser (https://www.deciphergenomics.org/).

A heatmap plot of methylation profiles was generated by plotting normalized beta-values (normalized methylation value by mean control value [(β-valuesample − β-valuecontrol_mean)/σ]. It was created with the ComplexHeatmap package (version 2.2.0). Hierarchical clustering was performed using a complete-linkage method by the ‘hclust’ option, and this unsupervised clustering result was then displayed as a dendrogram above the top annotation bar in the heatmap. A scatter plot provided detailed information about methylation patterns. More specifically, by comparing the methylation beta-values of individuals with the mean values of ±3 standard deviation confidence interval of healthy individuals, a significant gain or loss of methylation could be identified. A clustering plot was created using a PCA algorithm based on pre-processed beta-values (not normalized) in order to remove potential bias from normalized data. PCA (unsupervised clustering) was performed with the ‘prcomp’ option in R (v.3.6.3).

## Supplementary Material

Copy_of_Supplementary_Table_124567_updated0305_ddad079

supplementary_Table_3_8_9_ddad079

supp_figure_1_ddad079

supp_figure_2_ddad079

supp_figure_3_ddad079

supp_figure_4_ddad079

supp_figure_5_ddad079

supp_figure_6_ddad079

Supplementary_Figure_1-6_LEGENDS_ddad079
