# Supplementary material for: Epigenotype–genotype–phenotype correlations in SETD1A and SETD2 chromatin disorders
Source: Hum Mol Genet. 2023 May 11;32(22):3123–34. doi: 10.1093/hmg/ddad079 (PMC10630252; doi:10.1093/hmg/ddad079)
Supplement: supplementary_Table_3_8_9_ddad079 [file supplementary_table_3_8_9_ddad079.docx]

**Supplementary tables**

**Excel: [Supplementary Table 1,2]** Non-redundant DMPs using caret package

**[Supplementary Table 3]** Summary of the number of significant DMPs and ClinGen information

**Excel: [Supplementary Table 4,5,6]** Excel file with genotype-phenotype information raw data including CpG Island, DMB location for *SETD2*-1740 (sup4), *SETD2*-LLS (sup5), *SETD2* both (sup6)

**Excel: [Supplementary Table 7]** Comparison of the episignatures from Levy et al. (4,13)

**[Supplementary Table 8]** KMT (for validation analysis) samples variant details

**[Supplementary Table 9]** Number of sequencing coverage and mapped reads

**[Supplementary Table 3] Summary of the number of significant DMPs and ClinGen information**

|  | Total DMPs  (ncpgisland/nDMB) | genes |
| --- | --- | --- |
| SETD2-1740 (n=6) | 7566 (281/62) | 362 |
| SETD2-LLS (n=4) | 778 (34/8) | 43 |
| SETD1A LoF (n=6) | 7 (1/0) | 1 |
| SETD2-1740 + SETD2-LLS  (n=10) | 135 (5/1) | genes are annotated only for (one group vs controls), not for comparative analysis |
| SETD2-1740 + SETD2-LLS +  SETD1A (n=12) | 0 | genes are annotated only for (one group vs controls), not for comparative analysis |

Disease relation (number of phenotype MIMs reported in this region): possible clinical interpretation for genes related to disrupted methylated area from Decipher website.

**[Supplementary Table 8] *DYT-KMT2B* and KS1 patient variant details (from Lee et al. (5))**

| Patient ID | Disorder | Type | Age* | Gender | Mutation | Protein |
| --- | --- | --- | --- | --- | --- | --- |
| *KMT2B*_1  (DYT107P) | Early-onset Dystonia (DYT28) | proband | 41 | F* | c.3642+5G>A | Intron 11, donor splice site lost |
| *KMT2B*_2  (DYT130P) |  | proband | 31 | M* | c.3147_3160delGGGAGTGGGGGGC | p.Gly1050Profs*33 |
| *KMT2B*_3  (DYT52P) |  | proband | 4 | F | c.4688del | p.Ala1563Aspfs*83 |
| *KMT2B*_4  (DYT69P) |  | proband | 20 | F | c.3602delC | p.Pro1201Argfs*154 |
| *KMT2B*_5  (DYT78P) |  | proband | 29 | F | c.2137dupA | p.Thr713Asnfs*4 |
| *KMT2B*_6  (DYT79P) |  | proband | 42 | F | c.6439C>T | p.Gln2147* |
| *KMT2B*_7  (DYT80P) |  | proband | 24 | F | c.1656dupC | p.Lys553Glnfs*46 |
| *KMT2B*_8  (DYT81P) |  | proband | 12 | F | c.5658delC | p.Ser1887Profs*8 |
| *KMT2B*_9  (DYT98P) |  | proband | 23 | M | c.1107dupA | p.Glu370Argfs*19 |
| *KMT2B*_10  (DYT158P) |  | proband | 15 | M | c.3143_3149del | p.Gly1048Glufs*132 |
| *KMT2D*_1  (10002815) | Kabuki syndrome Type 1 | proband | 11 | M | c.14424delC | p.Ser4808Argfs*2 |
| *KMT2D*_2  (10004000) |  | proband | 35 | M | c.14946G>A | p.Trp4982* |
| *KMT2D*_3  (10004089) |  | proband | 33 | M | c.8488C>T | p.Arg2830* |
| *KMT2D*_4  (10007) |  | proband | 27 | F | c.14710C>T | p.Arg4904* |
| *KMT2D*_5  (10012157) |  | proband | 15 | M | c.12179_12182deICTGA | p.Thr4060Asnfs*4 |
| *KMT2D*_6  (31210) |  | proband | 34 | M | c.16412G>C | p.Arg5471Thr |
| *KMT2D*_7  (77428) |  | proband | 12 | F | c.8727_8730deIAAGT | p.Ser2910Argfs*32 |
| *KMT2D*_8  (85546) |  | proband | 25 | M | c.13895delC | p.Pro4632Hisfs*8 |
| *KMT2D*_9  (9005905) |  | proband | 11 | F | c.5527dupA | p.Thr1843Asnfs*5 |
| *KMT2D*_10  (9901174) |  | proband | 32 | M | c.14485dupG | p.Glu4829Glyfs*8 |

Variant details from 10 *DYT-KMT2B* and 10 KS1 patients. All annotation were aligned by GRCh19/hg19. *Age: Age of sample was acquired *F: Female *M: Male

**[Supplementary Table 9] Number of sequencing coverage and mapped reads**

| Sample | Total reads processed | Number of mapped reads (bismarkcov) |
| --- | --- | --- |
| SETD2-R1740Q-P1 | 60,319,221 | 11263225 |
| SETD2-R1740Q-P2 | 24,097,045 | 9452045 |
| SETD2-R1740W_P1 | 60,164,011 | 11017323 |
| SETD2-R1740W-P2 | 88,235,499 | 15229459 |
| SETD2-R1740W-P3 | 31,338,472 | 10693188 |
| SETD2-R1740W-P4 | 24,094,197 | 9689530 |
| SETD2-LLS-P1 | 26,093,490 | 9002514 |
| SETD2-LLS-P2 | 94,501,166 | 15638483 |
| SETD2-LLS-P3 | 57,192,947 | 16618676 |
| SETD2-LLS-P4 | 40,300,754 | 9941076 |
| SETD1A-P1 | 57,885,183 | 11917649 |
| SETD1A-P7 | 55,501,974 | 9133084 |
| SETD1A-P2 | 6,899,969 | 10459484 |
| SETD1A-P3 | 29,324,794 | 15866548 |
| SETD1A-P6 | 43,334,282 | 13333502 |
| SETD1A-P4 | 35,681,964 | 6420815 |
| SETD1A-P5 | 21,026,427 | 15080829 |
