# Supplementary figures and images for: Epigenotype–genotype–phenotype correlations in SETD1A and SETD2 chromatin disorders
Source: Hum Mol Genet. 2023 May 11;32(22):3123–34. doi: 10.1093/hmg/ddad079 (PMC10630252; doi:10.1093/hmg/ddad079)

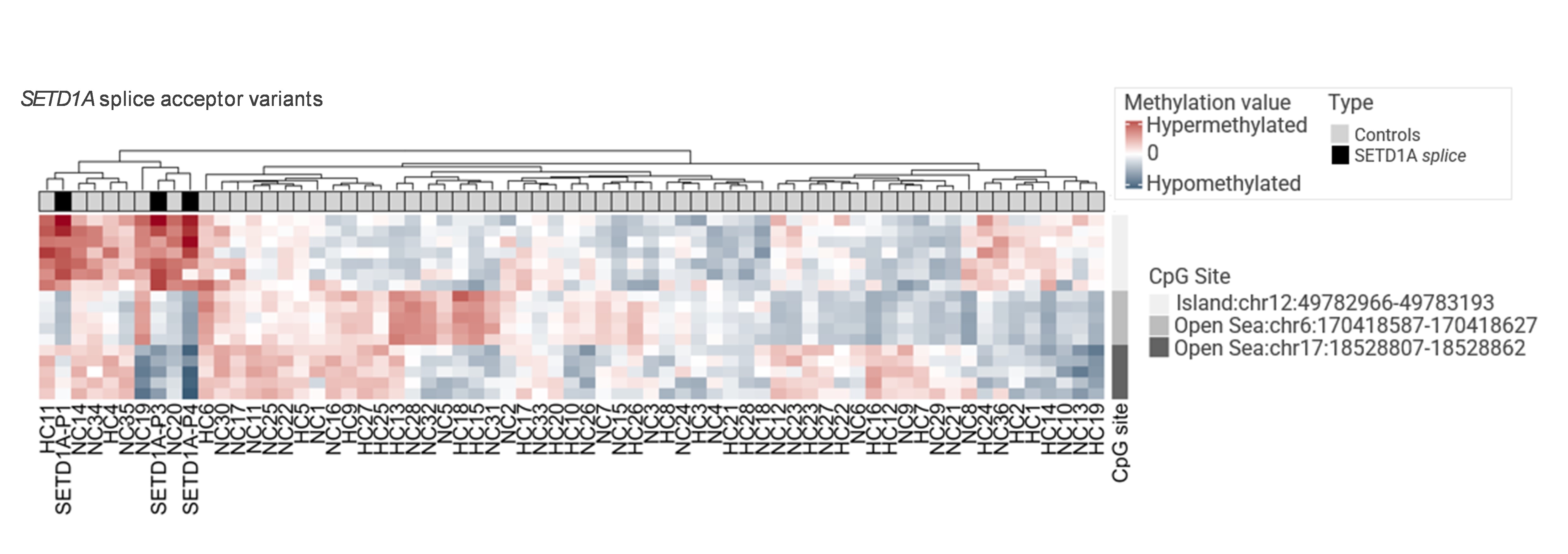

Supplement: supp_figure_1_ddad079 [file supp_figure_1_ddad079.jpeg]

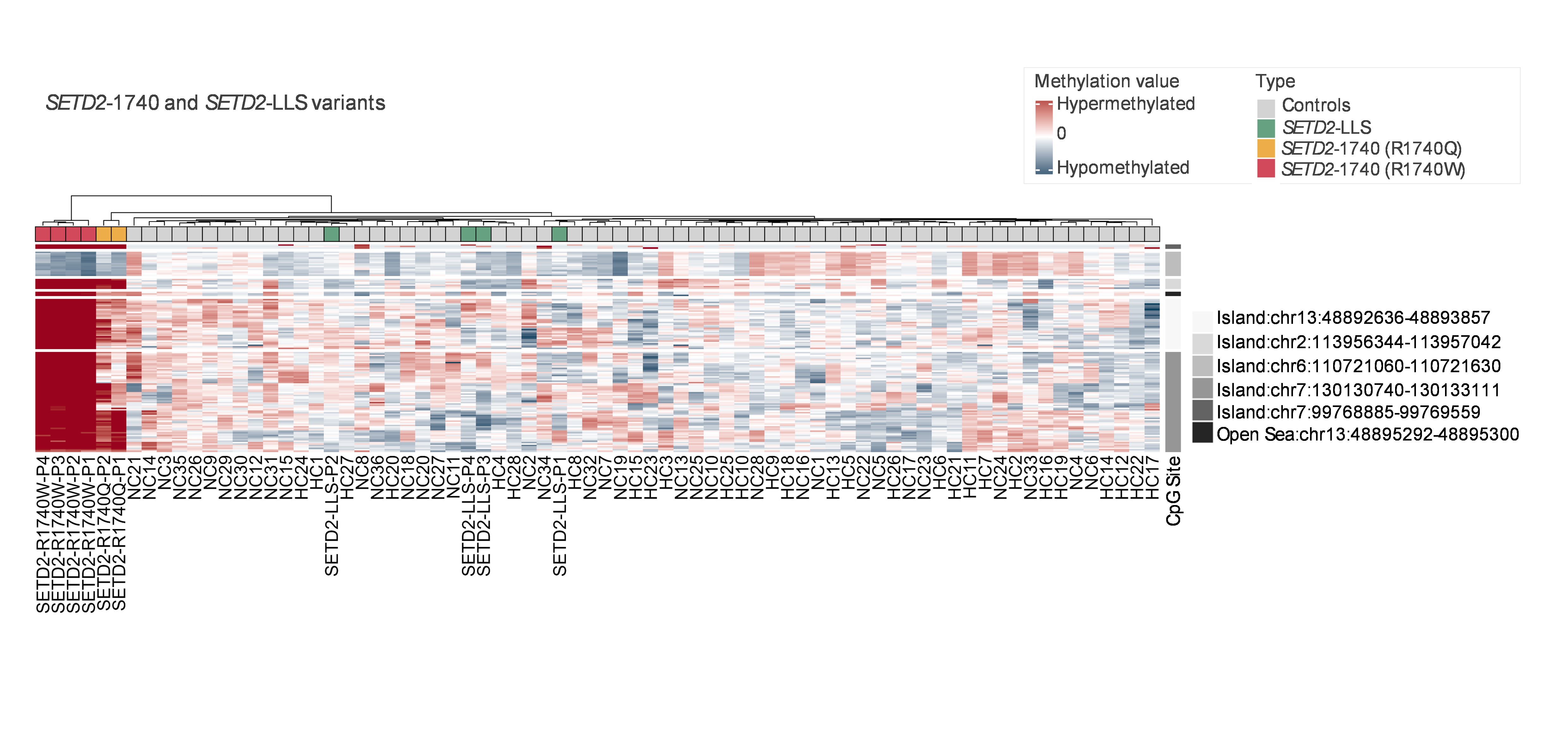

Supplement: supp_figure_2_ddad079 [file supp_figure_2_ddad079.jpeg]

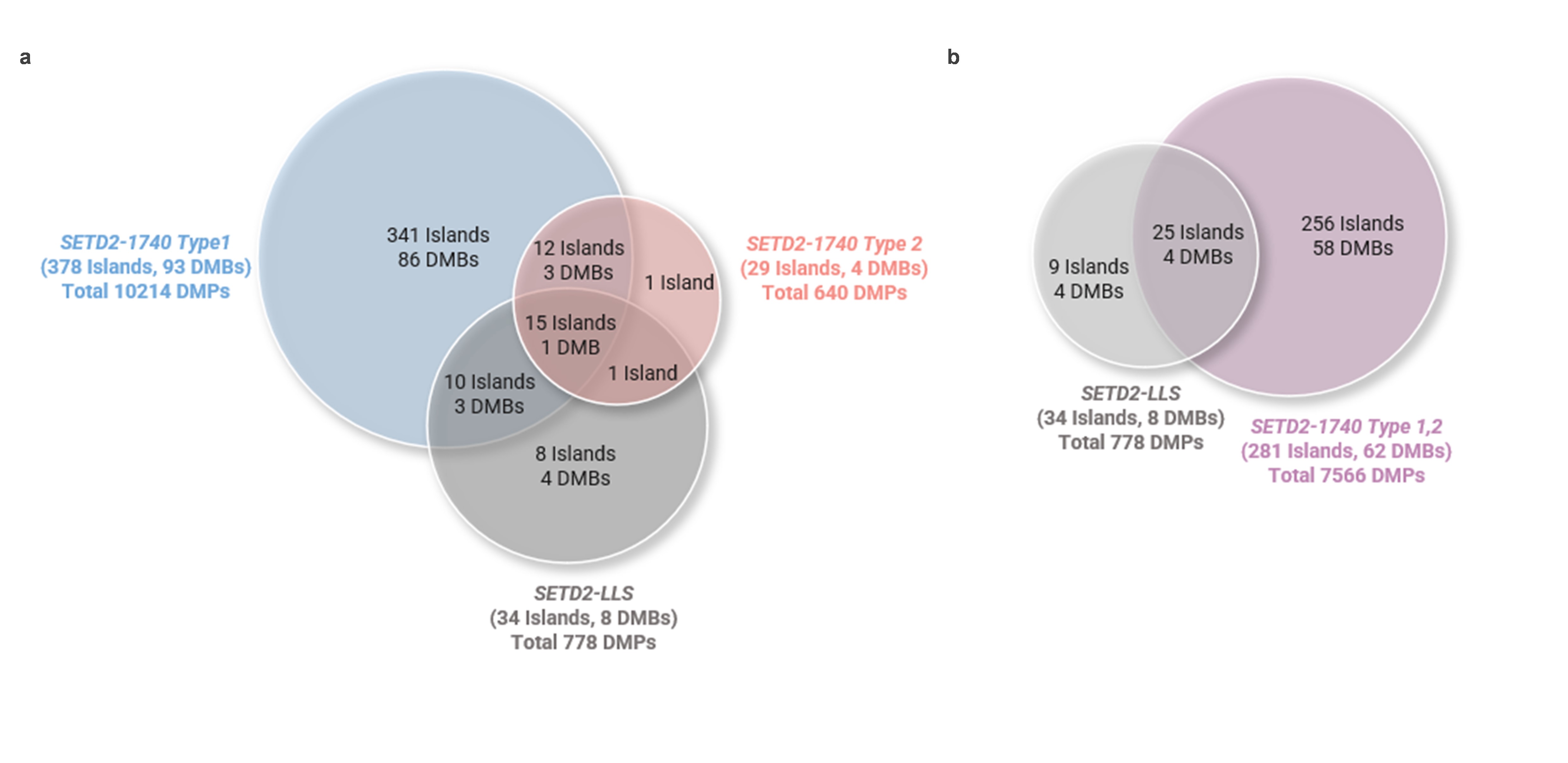

Supplement: supp_figure_3_ddad079 [file supp_figure_3_ddad079.jpeg]

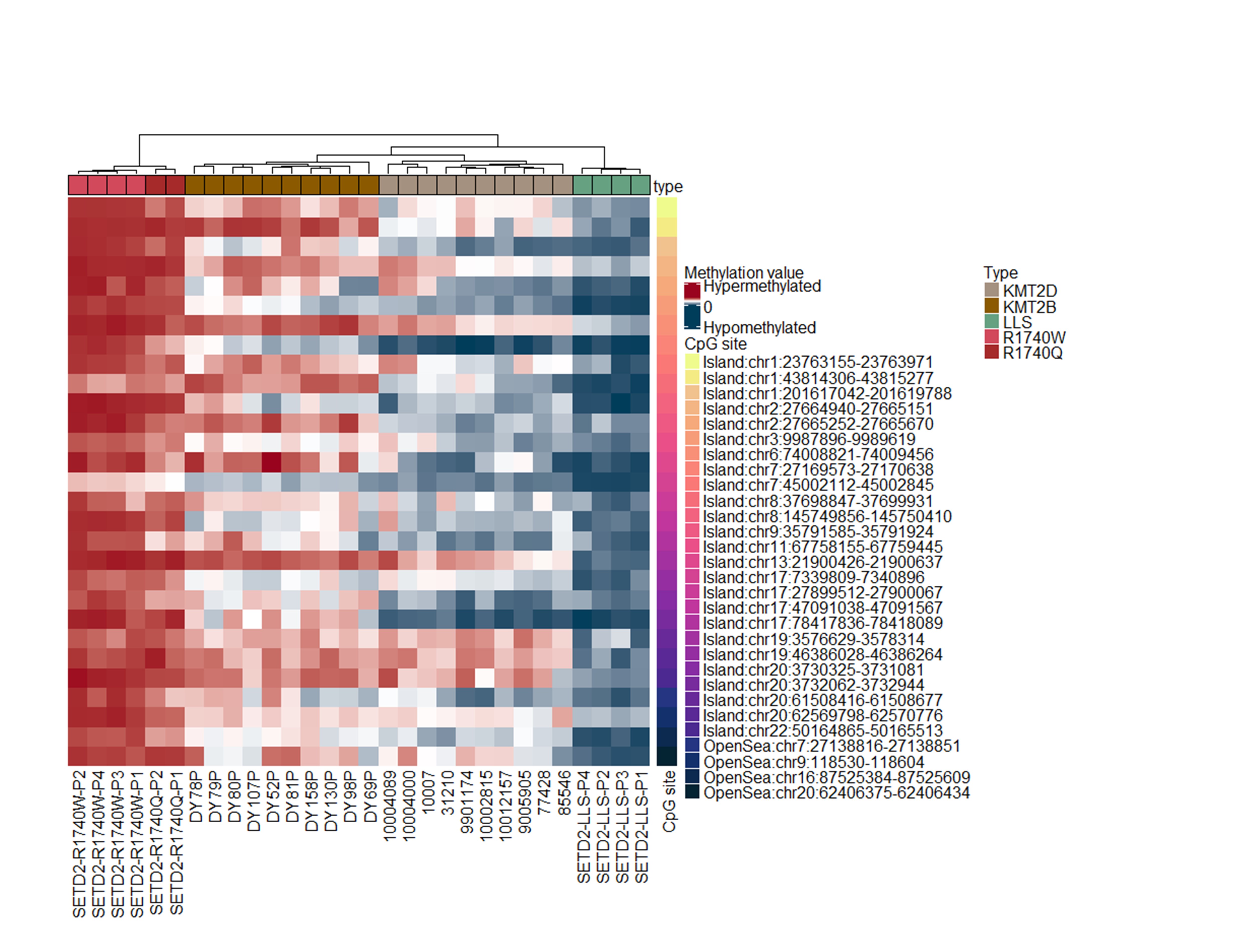

Supplement: supp_figure_4_ddad079 [file supp_figure_4_ddad079.jpeg]

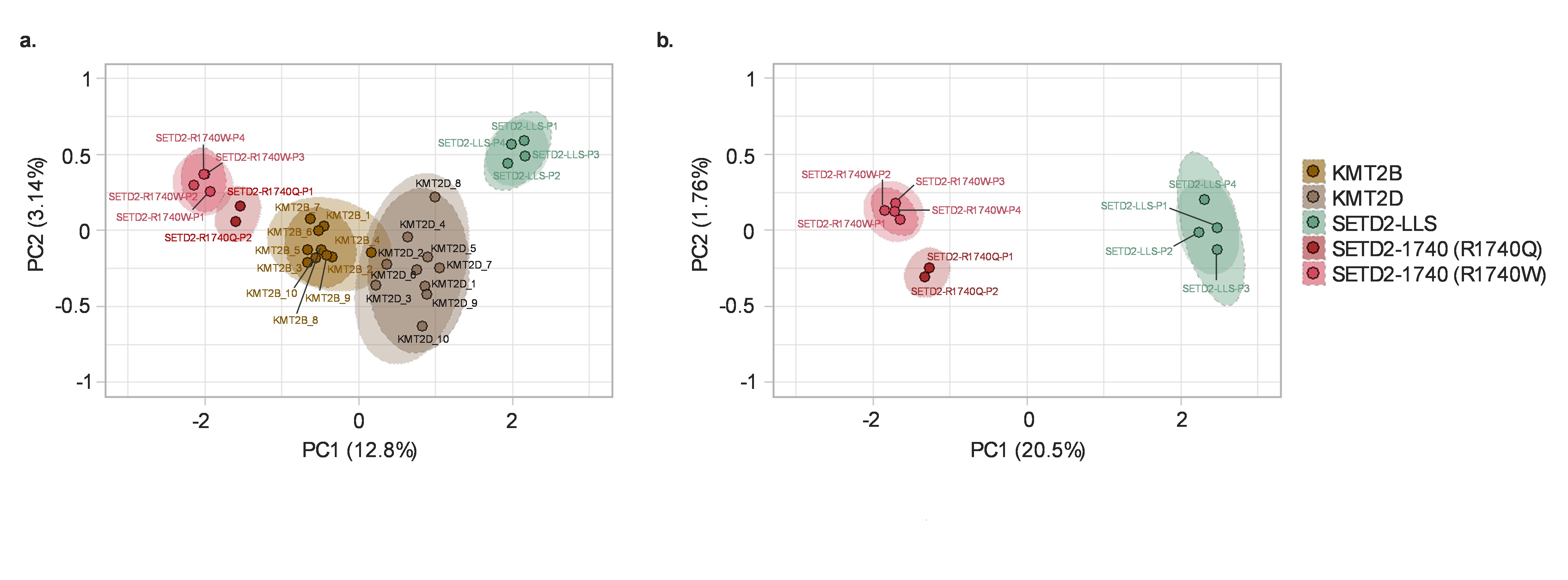

Supplement: supp_figure_5_ddad079 [file supp_figure_5_ddad079.jpeg]

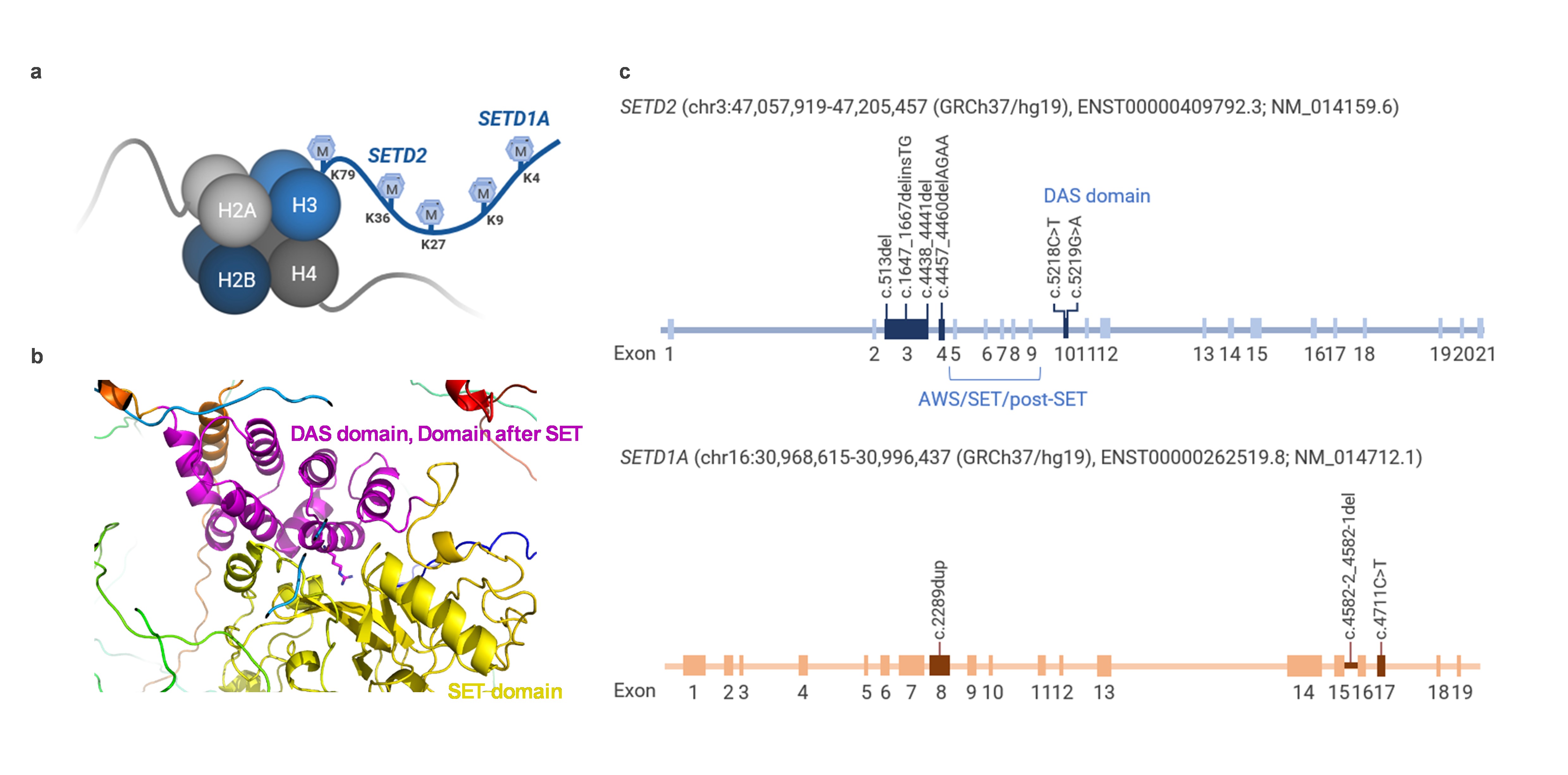

Supplement: supp_figure_6_ddad079 [file supp_figure_6_ddad079.jpeg]
