# Supplementary material for: Epigenotype–genotype–phenotype correlations in SETD1A and SETD2 chromatin disorders
Source: Hum Mol Genet. 2023 May 11;32(22):3123–34. doi: 10.1093/hmg/ddad079 (PMC10630252; doi:10.1093/hmg/ddad079)
Supplement: Supplementary_Figure_1-6_LEGENDS_ddad079 [file supplementary_figure_1-6_legends_ddad079.pdf]

Supplementary Figure Legends

[Supplementary Figure 1] The methylation profiling of *SETD1A* samples (n=3) with the recurrent splice-acceptor variant

[Supplementary Figure 2] Comparative analysis of *SETD2*-1740 and *SETD2*-LLS samples

[Supplementary Figure 3] Venn diagram of CpGs, DMBs and CpG islands for three groups of *SETD2* patients

[Supplementary Figure 4] Unsupervised clustering validation of selected 29 DMBs with other NDDs (*KMT2D*-related Kabuki syndrome and KMT2B-related childhood onset dystonia samples)

[Supplementary Figure 5] PCA Clustering of selected 29 DMBs

[Supplementary Figure 6] Locations of the *SETD1A* and *SETD2* variants on histone proteins

[Supplementary Figure 1] The methylation profiling of *SETD1A* samples (n=3) with the recurrent splice-acceptor variant

As a result of the methylation profiling of 3 *SETD1A* splice mutations, only 17 significant DMPs were identified, including two DMBs and one CpG island. The 17 DMPs, however, were unable to distinguish 3 splice mutation samples from control groups, indicating that *SETD1A* splice mutation samples did not exhibit a clear methylation episignature**.**

[Supplementary Figure 2] Comparative analysis of *SETD2*-1740 and *SETD2*-LLS samples

A combined methylation analysis of 10 patients (6 *SETD2*-1740 and 4 *SETD2*-LLS) revealed that 135 significant DMPs including 1 DMB and 5 CpG islands. Of the 135 DMPs detected, 17 were hypomethylated and 118 were hypermethylated in *SETD2*-1740, compared with 81 hypomethylated and 54 hypermethylated in *SETD2*-LLS. These numbers were calculated based on average normalised methylation value of each patient cohort (n=6, n=4, respectively). These 135 DMPs were able to distinguish between *SETD2*-1740 type1 and type2 and healthy controls, but not between *SETD2*-LLS group and controls, suggesting *SETD2*-LLS has a weaker methylation signature than SETD2-1740.

[Supplementary Figure 3] Venn diagram of CpGs, DMBs and CpG islands for three groups of *SETD2* patients

Based on the analysis of the *SETD2*-1740 Type1 and Type 2 groups separately, a total of 10,214 DMPs are detected in the *SETD2*-1740 Type 1 group, and 640 DMPs are detected in the *SETD2*-1740 Type 2 group. In a combined analysis of Type 1 and Type 2 mutations, 7,566 DMPs are identified as significant. In the *SETD2*-LLS group, 778 significant DMPs are identified. **S4a)** All three *SETD2* subgroups have 15 CpG islands and one DMB in common. The majority of significant DMPs in *SETD2*-1740 type 2 are also significant in type 1. Similarly, over half of the DMPs in *SETD2*-LLS overlap with *SETD2*-1740. **S4b)** Of the 7,566 significant DMPs in the *SETD2*-1740 Type1 and 2 cases, 513 DMPs (including 25 Islands and 4 DMBs) were also significant in *SETD2*-LLS group. In the *SETD2*-LLS group, over half of the significant CpG islands and DMBs overlapped with the *SETD2*-1740 group.

[Supplementary Figure 4] Unsupervised clustering validation of selected 29 DMBs with other NDDs (*KMT2D*-related Kabuki syndrome and KMT2B-related childhood onset dystonia samples)

The heatmap displays the methylation episignatures for 29 *SETD2* DMBs comparing to *KMT2B* and *KMT2D* cohort. Unsupervised clustering result showed that these 29 DMBs successfully discriminate SETD2 NDDs from other NDDs.

*KMT samples variant details are in Supplementary Table

[Supplementary Figure 5] PCA Clustering of selected 29 DMBs

Unsupervised PCA clustering results for *SETD2* cohort and the comparison with other NDDs (*KMT2B* and *KMT2D* cohort from our previously published paper (5). **S6a)** The analysis revealed that all 5 groups, including subgroup of *SETD2*-1740 subgroup, exhibited distinct episignatures and were clearly separable. **S6b)** Furthermore, it was observed that the *SETD2*-1740 group and *SETD2*-LLS group exhibited distinct profiles and were well distinguishable from one another.

[Supplementary Figure 6] Locations of the *SETD1A* and *SETD2* variants on histone proteins

S1a) Locations of *SETD2* and *SETD1A* on H3K36 and H3K4 histone proteins **S1b)** Protein structure of SETD-domain and DAS domain **S1c)** Locations of *SETD2* and *SETD1A* variants
